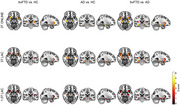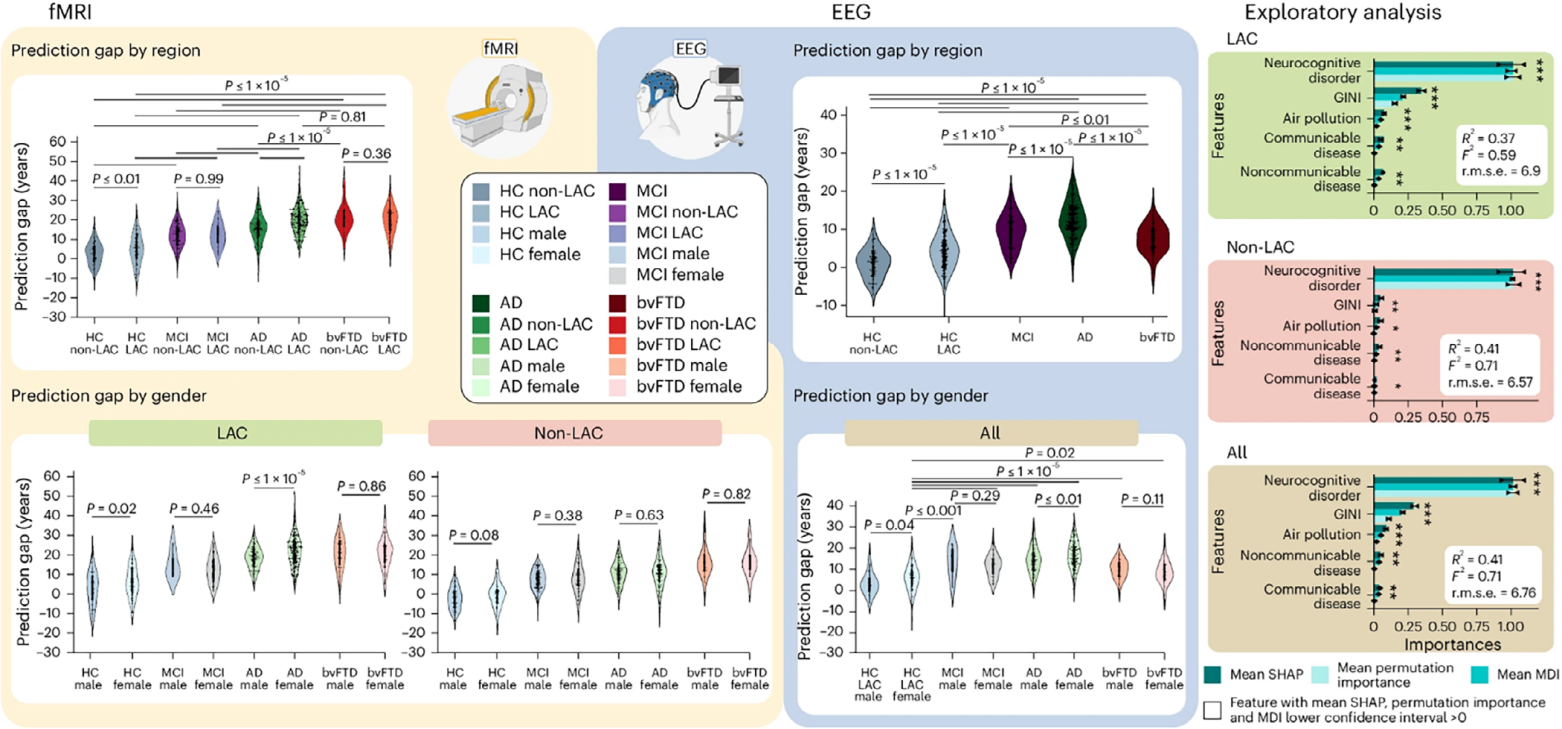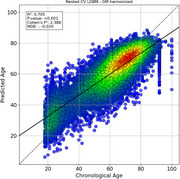# Personalized AI Models for Accelerated Aging

**DOI:** 10.1002/alz70856_099932

**Published:** 2025-12-24

**Authors:** Sebastian Moguilner, Sandra Baez, Hernan Hernandez, Agustina Legaz, Robert Whelan, Agustin Ibanez

**Affiliations:** ^1^ Massachusetts General Hospital, Boston, MA, USA; ^2^ Latin American Brain Health Institute (BrainLat), Universidad Adolfo Ibáñez, Santiago, Región Metropolitana de Santiago, Chile; ^3^ Universidad de los Andes, Bogotá, Colombia, Bogota, Bogota, Colombia; ^4^ Global Brain Health Institute (GBHI), Trinity College Dublin, Dublin, Dublin, Ireland; ^5^ Latin American Brain Health Institute (BrainLat), Universidad Adolfo Ibañez, Santiago, Chile; ^6^ Cognitive Neuroscience Center (CNC), Universidad de San Andrés, Buenos Aires, Buenos Aires, Argentina; ^7^ Global Brain Health Institute (GBHI), University of California San Francisco (UCSF); & Trinity College Dublin, Dublin, Ireland

## Abstract

**Background:**

The current understanding of brain aging and dementia diagnostics relies heavily on standardized data from homogeneous populations in the Global North, which creates challenges in assessing diverse populations. Critical gaps exist in understanding how geographical, socioeconomic, and demographic factors impact accelerated brain aging across global populations, particularly in underserved regions.

**Method:**

We developed three approaches: (1) A computer‐vision DenseNet classifier analyzing raw structural MRI data from 3,000 participants with behavioral variant frontotemporal dementia (bvFTD), Alzheimer's disease (AD), and healthy controls; and (2) A graph‐based deep learning architecture analyzing High‐Order Interactions from fMRI and EEG data from 5,306 participants across 15 countries to study the difference between predicted age and chronological age (brain‐age gaps, BAGs); and (3) By analyzing associations between BAGs and exposomes using nonlinear Generalized Additive Models (GAMs) in a multimodal approach combining atrophy, fMRI and EEG.

**Result:**

(1) The DenseNet classifier demonstrated robust performance across standardized 3T and non‐standardized 1.5T clinical images, with disease‐specific patterns identified in key brain regions of AD and FTD pathology. (2) The BAGs analysis revealed significantly older brain ages in Latin American and Caribbean (LAC) populations compared to non‐LAC regions. Structural socioeconomic inequality, pollution, and health disparities strongly predicted increased BAGs, particularly in LAC. We observed larger BAGs in females within LAC control and AD groups and an ascending BAG pattern from healthy controls to mild cognitive impairment to AD. (3) Our GAM results so far showed significant associations between BAGs and various factors including Human Development Index, economic equality, air pollution, and communicable disease death ratios, with all models showing high statistical significance (*p* <0.0001). A meta‐model combining GAM predictions demonstrated enhanced predictive power compared to individual models, suggesting that aggregate‐level environmental factors have meaningful effects on brain health globally.

**Conclusion:**

These findings can provide scalable solutions to address accelerated aging disparities, particularly in underserved settings. GAM multimodal meta‐models increase the performance of BAGs estimations. Personalized AI tools can study accelerated brain aging trajectories and their modifiable risk factors.